# Public Health

**Published:** 1874-12

**Authors:** 


### MISCELLANEOUS

### PUBLIC HEALTH.

### PROPOSED BUREAU OF VITAL STATISTICS IN OHIO.

At the annual session of the Ohio State Medical Society, held in Toledo, in June last, Dr. Jones, of Toledo, as Chairman of the "Committee on State Medicine and Public Hygiene," read a very able paper, which attracted much notice from his professional brethren, and was printed in the proceedings of the Society. The effect of the paper was to cause a committee to be appointed, of which Dr. Jones was made chairman, to take into consideration plans by which the vital statistics could be collected from year to year by competent persons, and given to the public for the benefit of the profession and the people. The Committee has prepared the following memorial to the Legislature, which sets forth the wishes of the Society:

To the Hon. the General Assembly of the State of Ohio:

At the annual meeting of the Ohio State Medical Society, held at Toledo, the undersigned were appointed a Committee to memorialize your Honorable body, for the passage of a law. creating a Bureau of Health, with a Sanitary Commissioner.

In urging upon your Honorable body the passage of such a law, our Society believed that the Medical profession would co-operate in making it contribute not only to the happiness of longevity of the people of the State, but by this means, many of the causes of disease (which will forever elude individual effort to trace them out) may be made plain, so that they may be avoided, and the people of the State relieved of much of the cost and suffering now entailed upon them, from the want of Sanitary knowledge.

W. W. Jones, M. D., Toledo,
E. H. Hyatt, M. D., Dalaware,
John A. Murphy, M. D., Cincinnati,
J. W. Scott, M. D., Cleveland,
John Bennett, M. D., Cleveland.

Com. of the Ohio State Medical Society.

December 9th, 1874.

Accompanying this is the following bill, which has been drawn up in accordance with the ideas of the Committee upon the subject, and offered in the Senate by Judge Potter:

### A BILL

To provide for the appointment of a Health Commissioner, and prescribe his duties.

Section 1. *Be it enacted by the General Assembly of the State of Ohio*, That there shall be appointed by the Governor, by and with the advice and consent of the Senate, within —— days after the passage of this act, a person to be styled the Commissioner of Health, who shall be a regular physician in good standing in his profession, who shall hold his office for three years, and until his successor is appointed and qualified. The person so appointed shall be an elector of this State, and shall keep an office in the State House. In case of vacancy by death, resignation, removal from the State or otherwise, the Governor shall fill the vacancy and report the name of such appointee to the Senate, if in session, if not, within ten days after the commencement of the next session, who, by the consent of the Senate, shall hold his office for the unexpired term of three years, or until his successor is appointed and qualified; provided, that if at any time the Governor shall become satisfied that the Commissioner is inefficient or derelict in the discharge of the duties of the office, he as hereby authorized and required, by and with the consent of the Senate, if it be in session, to remove said Commissioner from office; and if the Senate be not in session, to suspend him from the duties of said office, temporarily filling the vacancy as provided for in this section, and report the facts to the Senate when in session.

Sec. 2 Before entering upon the discharge of the duties of the office, said Commissioner shall take the usual oath or affirmation required by the constitution of the State. He shall receive for his services the sum of three thousand dollars per annum, and be furnished with an office, office furniture, necessary blanks, stationery and postage, at the expense of the State. He shall have power to employ a clerk to perform such duties as may be assigned by him, to be paid out of the State treasury, at the rate of twelve hundred dollars per annum.

SEC. 3. It shall be the duty of the Commissionor to supervise the interest of the health and life of the people of the State, to study and collect the vital statistics of this State, and endeavor to make intelligent and profitable use of the collected records of births, marriages, deaths and sickness among the people; to make sanitary investigations and enquiries respecting the causes of disease, and especially of epidemics, the causes of mortality, and the effects of localities, employments, conditions, ingesta, habits and circumstances on the health of the people. He shall when required, or when they deem it best, advise officers of the government, benevolent institutions, or other States, city, county and township boards in regard to the location, drainage, water supply, disposal of excreta, heating or ventilation of any public institution or building, and shall, from time to time, recommend standard works upon hygiene and sanitary science for the use of the schools of the State. He shall, so far as practicable, collect and have the custody of all books, papers and documents relating to the subject of health which may come into his possession through exchanges and correspondence with health organizations in this and other States, prepare such blank forms and returns as may be necessary, and forward them to the several boards of health or physicians throughout the State, so as to select statistics as to the causes of disease.

SEC. 4. For the purpose of affording the Health Commissioner better advantages for obtaining knowledge important to be incorporated with that collected through special investigations and from other sources, it shall be the duties of all officers of this States, all boards of health, officers or physicians, of mining, railroad, or other corporations or boards, organized under the laws of this State, to report to him any information bearing upon the public health which may be requested by him, for the purpose of enabling him better to perform the duties of the office in collecting and distribuuseful knowledge upon this subject.

SEC. 5. He shall make an annual report to the Governor, to be transmitted to the General Assembly, which shall embody the vital statistics and facts which he may be able to obtain, and his deductions therefrom; knowledge respecting the causes of disease, and useful information on the subject of hygiene for the purpose of dissemination among among the people.

